# Improving apoptotic responses to targeted therapy

**DOI:** 10.18632/oncotarget.1261

**Published:** 2013-08-06

**Authors:** Rizwan Haq, David E. Fisher

**Affiliations:** Department of Dermatology, Massachusetts General Hospital; Department of Dermatology, Massachusetts General Hospital

Comprehensive genomic analyses, such as genome sequencing, transcriptome analyses and functional screens, have identified oncogenic ‘driver’ mutations in a variety of cancers [1]. Correspondingly, small molecule inhibitors targeting these mutant oncoproteins have demonstrated promise in the clinic. However, most, if not all, targeted therapies usually fail to eradicate disease. The most likely explanations are either insufficient inhibition of the driver oncoprotein or the existence of genetic or epigenetic alterations in cancer cells that limit the effectiveness of targeted therapy. Overcoming such intrinsic resistance of cancer cells may therefore improve the efficacy of targeted therapy. In melanoma, the most common targetable genetic alteration is oncogenic activation of the protein kinase BRAF, leading to its constitutive activity and downstream MAPK signaling [2, 3]. Vemurafenib, an ATP-competitive BRAF inhibitor has been approved for use in patients and improves overall survival compared to standard of care chemotherapy. Similar to many other targeted therapies, it prolongs survival only on average several months compared to chemotherapy. We recently identified a lineage-specific anti-apoptotic factor, BCL2A1, that is recurrently amplified in approximately one third of human melanomas [4]. *BCL2A1* is a member of the five-gene anti-apoptotic BCL2 family, each which has been shown to be dysregulated in several cancer types [5]. We reported that *BCL2A1* expression is restricted to the melanocyte lineage by virtue of its direct regulation by the melanocyte master regulator and melanoma oncogene, MITF. We also showed that the apoptotic response to BRAF inhibitors could be significantly enhanced by suppression of *BCL2A1*. Importantly, a small molecule called obatoclax, which inhibits BCL2 family members including Bcl2A1, enhanced the efficacy of BRAF inhibitors in vitro and in mouse models. Together with other reports in colon cancer or hematologic malignancies [6], these data indicate that combinations of targeted therapy along with small molecule inhibitors of the anti-apopototic family may have synergistic clinical utility. In addition to obatoclax, small molecule antagonists of BCL2 family members such as navitoclax (ABT-737) and ABT-199, as well as others are in clinical development [7, 8]. Though evaluation of navitoclax has been subject to study in some cancer types, these drugs inhibit Bcl-2 and Bcl-XL, but not Bcl2A1 or Mcl1. Moreover high levels of *BCL2A1* and *MCL1* are typically associated with resistance to such agents [9]. Thus, treatment of patients with anti-apoptotic drug combinations will need to be individualized depending on the key apoptotic proteins that are dysregulated in each tumor.

Since the BCL-2 family can be regulated by targeted therapy and chemotherapy, it is likely that dynamic changes in their expression and interactions with other BCL-2 family members will necessitate innovative approaches to develop reliable biomarkers. The development and validation of such biomarkers are areas of intense investigation, and may permit rational combination therapies using targeted therapy and anti-apoptotic inhibitors. The reward of successful cancer cell killing will, it is hoped, lead to improved outcomes for our patients.

## References

[1] Eifert C, Powers RS (2012). Nat Rev Cancer.

[2] Chapman PB (2011). N Engl J Med.

[3] Davies H (2013). Proc Natl Acad Sci U S A.

[4] Haq R (2013). Proc Natl Acad Sci U S A.

[5] Beroukhim R (2010). Nature.

[6] Cragg MS (2008). J Clin Invest.

[7] Souers AJ (2013). Nat Med.

[8] Oltersdorf T (2005). Nature.

[9] Yecies D (2010). Blood.

